# The Impact of Emotional Labor on User Stickiness in the Context of Livestreaming Service—Evidence From China

**DOI:** 10.3389/fpsyg.2021.698510

**Published:** 2021-07-08

**Authors:** Yunxia Shi, Chunhao Ma, Yuxin Zhu

**Affiliations:** ^1^School of Management, Shandong Technology and Business University, Yantai, China; ^2^School of Management, Huazhong University of Science and Technology, Wuhan, China

**Keywords:** livestreaming service, emotional labor, user stickiness, immersion theory, regulatory focus theory

## Abstract

Improving the user stickiness becomes increasingly valued, due to the severe user churn of livestreaming services. Previous studies pay much attention to the influencing factors of technology on user stickiness, ignoring the emotional factors. This study examined the impact of the emotional labor of network anchors (deep acting vs. surface acting) on user stickiness in the context of livestreaming service. We extended prior findings in three ways. The results of Study 1 (i.e., questionnaire method, 305 livestreaming users, and 56.4% females) demonstrated that the emotional labor of network anchor positively influenced user stickiness, and immersion experience plays a mediating role. The results of Study 2 (i.e., situational simulation method, 203 volunteers, and 54.09% females) demonstrated that the deep acting strategies of emotional labor had a stronger effect when compared with surface acting strategies. The results of Study 3 (i.e., situational simulation method, 235 volunteers, and 51.9% females) demonstrated that the effect of emotional labor on user stickiness was stronger for the users with prevention focus compared with promotion focus. Based on the perspective of emotional labor, this study extends the previous research on user stickiness and is valuable for guiding the practice of livestreaming services.

## Introduction

Livestreaming services have a profound impact on social life, such as the rapid development of online courses and e-commerce livestreaming, which attract more and more attention. The competition among platforms offering livestreaming services becomes fierce. Users have more rights to select and switch among the similar livestreaming platforms, resulting in the problems of user churn. How to retain users becomes a hot topic. User stickiness is used to measure the ability of online service providers to retain users (Zott et al., 2000). However, previous studies pay much attention to the technological factors on user stickiness, such as perceived usefulness, ignoring the effect of emotional factors. In fact, most people choose to accept livestreaming services for hedonic values such as satisfying emotional needs (Hsu et al., 2020). As the core element of livestreaming services, the emotional labor of network anchors is an important source to satisfy the emotional experience of users (Mardon et al., 2018). According to the immersion theory, in an interactive online environment, a good emotional experience can induce an immersive experience that refers to a state in which an individual is fully engaged in an activity (Jennett et al., 2008). Users with high immersion experience are highly involved and filled with joy and excitement inside, ignoring the passage of time. Previous studies have shown that immersive experiences facilitate user stickiness (Fang et al., 2019). However, can emotional labor affect the immersion experience? Does the immersion experience play a mediating role between emotional labor and user experience? These questions are still unclear.

The generation of the immersion experience of the user depends on his/her traits. Therefore, we distinguished users with different individual traits to examine the differentiation in their behavior (i.e., user stickiness). Individual traits can be divided into the promotion focus and the prevention focus, according to the regulatory focus theory (Higgins, 1997). Individuals with a promotion focus are more concerned with positive information (e.g., gains and hedonic value) and more likely to engage in positive behaviors. Individuals with a prevention focus are more sensitive to negative information and behave more cautiously. We speculated that users with different regulatory focus have different feelings when perceiving the emotional labor of network anchors. They will have different degrees of immersion experiences. The user stickiness will be also different.

This study extends the previous studies in the following aspects: First, it explores the impact of emotional labor on user stickiness in the context of livestreaming, and for the issue of livestreaming, user stickiness has not received sufficient attention. Second, this study explains the mechanism of user stickiness based on the immersion theory and verifies the mediating role of immersion experience between emotional labor and user stickiness. Third, this study distinguishes the differentiation effects of individuals with different regulatory focus and explores the boundary conditions of the impact of emotional labor on user stickiness. Finally, many researchers suggest focusing on the perceptual perspective of emotional labor. This study redefined emotional labor from the perspective of the service recipient and developed the emotional labor theory to some extent.

The chapters are organized as follows: In the “Theoretical basis and research hypothesis” section, we reviewed the theory of emotional labor and presented our research model; in the “Hypothesis testing” section, we empirically tested the hypotheses through three studies; and in the “Discussion” section, we discussed the findings and gave theoretical contributions and practical insights.

## Theoretical Basis and Research Hypothesis

### Emotional Labor Theory

Hochschild (1983) first proposed the concept of emotional labor, describing the process in which service providers manage their own emotional expressions to create emotional states that meet the requirements of the organization. For example, a flight attendant should be smiling and helpful, a nurse should be sympathetic, and a funeral master should be careful to be serious and solemn (Ashforth and Humphrey, 1993; Gardner and Avolio, 1998; Grandey, 2003; Shapoval, 2019). In the process of emotional labor, surface acting and deep acting are the two strategies commonly adopted by service providers. The former refers to meet the requirements of emotional expression by suppressing real emotions (Katz-Navon et al., 2019). The latter refers to create the desired emotional state by adjusting the potential emotional state actively (Diefendorff et al., 2005; Chi and Grandey, 2019).

Research on emotional labor mostly discusses the relationship between staff and customers in the traditional frontline service industry, such as tourism, hospitality, and banking industry (Grandey and Melloy, 2017; Choi et al., 2019; Lechner and Paul, 2019). A few studies have examined the issue of emotional labor among members within organizations (Bhave and Glomb, 2013; Becker et al., 2017; Deng et al., 2020; Gabriel et al., 2020). With the development of the online service industry, the attention to emotional labor is gradually shifting from offline to online.

This study measures the emotional labor of network anchors from the perspective of user perception. The reasons are as follows: Emotional labor is a two-way interactive process. The perception of the recipient of the service is equally important (Grandey and Melloy, 2017). Groth et al. (2009) proposed that the difference between surface acting and deep acting is reflected not only in the cognitive adjustment process of the service provider but also in the recognition of emotions by the recipient of the service. Liu et al. (2019) explained the inconsistency in the perception of both parties in the process of emotional labor. The study suggested by Gong et al. (2020) further supports these views. Therefore, the emotional labor could be reported by the service recipient and the service provider. In other words, in our study, the users evaluate the emotional labor of the network anchors, and it is more objective and reasonable in terms of effective measurement. In this study, emotional labor is defined as the emotional effort perceived by users through observing the external performance of the network anchors (e.g., expression, voice, and movement).

In summary, this study improves the research related to emotional labor in the following two aspects. On the one hand, it further enriches the application of emotional labor theory in the context of online services and provides an empirical basis for research related to emotional labor in the context of livestreaming services (Mardon et al., 2018). On the other hand, although some researchers have called for the academic community to pay attention to the perception of service recipients in the process of emotional labor, however, such studies are still insufficient (Groth et al., 2009; Liu et al., 2019; Gong et al., 2020). This study examines how the emotional labor of network anchors affects user stickiness from the perspective of user perception.

### Perceived Emotional Labor and User Stickiness

User stickiness is an important indicator of the performance of online services (Rong et al., 2019). Li et al. (2006) defined user stickiness as the willingness of users to visit a website regularly and for a long period of time. Yan et al. (2020) regarded user stickiness as cognitive and affective, which made users use a specific item of service repeatedly.

Hsu et al. (2020) proposed that the motives of users for choosing livestreaming services include information acquisition, sociality, and entertainment needs and that users have stronger sociality and entertainment needs compared with information acquisition needs. Meng et al. (2020) found that the realization of hedonic value is more likely to increase the loyalty of users to livestreaming services. Users will stay longer if the emotional labor of the network anchor can satisfy the hedonic value (Holman et al., 2002; Jin and Oriaku, 2013; Zhang et al., 2017). Specifically, the network anchor shows the desired emotional state of the user with infectious words and physical expressions through the bullet–screen interaction, thus attracting users to keep watching. Zhang et al. (2017) examined that perceived enthusiasm can influence the formation of user stickiness through the mediating role of hedonic value. When the emotional labor is perceived, users feel delighted and generate positive emotions and perceptions. A high-intensity attachment relationship between network anchors and users was established through a process of positive emotional infection. In other words, the emotional labor of network anchor can satisfy the need of the user for an emotional experience. According to the self-determination theory, in order to continuously acquire this emotional need and hedonic value, users will extend the time of watching the live broadcast. Thus, the hypothesis is proposed as follows:

H1: User perceived emotional labor positively affects user stickiness.

Different network anchors may differ in their emotional performance. For example, some anchors adopt a more enthusiastic and sincere approach to interact with their audience (i.e., deep acting), while others may force themselves to serve with a smile simply to fulfill the job requirements (i.e., surface acting). We speculated that the two strategies have a differential impact on user stickiness. The following study is based on the emotions-as-social-information theory, which is analyzed and explained from the perspective of information processing. This theory suggests that emotional expressions carry a part of the social information in the process. Recipients influence their behavior through affective reactions to emotional expressions and inferential processes to information (Van-Kleef, 2009). In the traditional interpersonal interaction context, individuals tend to receive information through facial, voice, and action feedback (Hülsheger and Schewe, 2011). When it comes to the context of livestreaming service, users can also complete the information transfer process through the image display and voice tone of the network anchor on the screen (Meng et al., 2020). When the network anchor adopts a disguised emotional display such as a fake smile, users can easily detect and perceive it as a surface acting by analyzing the underlying information in the emotion (Yao et al., 2019). At this point, users may associate it with the personality qualities such as lying and insincerity, reducing the perception of service quality and thus weakening user stickiness (Tamilmani et al., 2019). In contrast, if the emotional state is naturally generated and the network anchor actively interacts with the user to create a feeling of intimacy, the user is more likely to perceive the deep acting (Hu et al., 2017). Anchors who adopt the deep acting strategy will be more infectious. A good emotional state leads to positive attitudes and behaviors of users. Therefore, users are more willing to receive livestreaming services repeatedly (i.e., to generate higher user stickiness). Based on the above-mentioned analysis, the hypothesis is proposed as follows:

H2: Deep acting has a stronger positive effect on user stickiness than surface acting.

### Mediating Effect of Immersive Experience

In the context of livestreaming service, users often enter a state of immersion and have an immersive experience, which is manifested as highly focused attention and loss of self-awareness (Jennett et al., 2008; Fang et al., 2019). Leung (2020) found that the act of watching livestreaming promotes hedonic value, which positively influences the generation of immersive experiences and thus increases the willingness of users to continue using it. In a word, users generate positive emotions while watching the livestream, filling with pleasure and satisfaction inside. According to the immersion theory, they expect this positive state to be maintained, thus increasing the motivation to keep watching the livestream (Csikszentmihaly, 1975). We speculated that the emotional labor of network anchor may positively affect the immersion experience of the user. For example, in the e-commerce-type livestreaming service, the network anchor will introduce the goods with enthusiasm, render the atmosphere with funny and humorous expressions, and answer all kinds of questions from users (Meng et al., 2020). This process gives users a sense of pleasure and satisfaction, and they are fully engaged in the live service, thus easily entering the immersion experience. Many studies have shown that users in an immersive experience are prone to positive behaviors, such as user loyalty and willingness to use the service continuously (Fang et al., 2019; Hudson et al., 2019). Zhang et al. (2017) found that satisfaction based on user experience is an important factor in forming stickiness. When entering the state of the immersion experience, individuals will get a good user experience and want to get this experience again in the future. Based on the above-mentioned analysis, the hypothesis is proposed as follows:

H3: Immersion experience plays a mediating effect role between perceived emotional labor and user stickiness.

### Moderating Effect of Regulatory Focus

There are differences in the perception of livestreaming services between different users. Thus, different behaviors may be exhibited. For example, some users are concerned about the authenticity and reliability of livestreaming services, while others are more concerned about entertainment and hedonic value. The regulatory focus theory can better explain these differences. Higgins (1997) argued that individual behavioral motivation is not only governed by hedonic principles but also generates different psychological adjustment activities when achieving different goal states. Individuals are classified into two types, namely, prevention focus and promotion focus. The former pays more attention to negative information such as risks, uses fine-grained processing of information, and thus behaves more cautiously (Beersma et al., 2013). The latter focuses more on hedonic values, takes heuristic processing of information, and treats things more inclusively (Amodio et al., 2004). Research has shown that in social interaction situations, individuals with prevention focus look for cues that may indicate a lack of security in the interaction and take risk-averse behaviors. In contrast, individuals with promotion focus do not pay attention to the above-mentioned cue information (Song and Qu, 2018). This study hypothesizes that different regulatory focus may lead to differential effects during the influence of emotional labor on user stickiness, and the reasons are explained in the following two paragraphs.

Chang et al. (2019) showed through a study of online community members that individuals with different regulatory focus feel differently about the emotions of others and thus make different judgments. Users with prevention focus are cautious and vigilant, prefer self-protection strategies, and are highly guarded against negative outcomes (Higgins, 2000). According to the emotional social information theory, the emotional information is analyzed meticulously by the users of prevention focus. They will be more likely to perceive risk when faced with an untrue expression of emotion from the network anchor, and the mistrust caused by this uncertainty may be passed on to the perception of things related to them (Friedman and Forster, 2001). For example, in the livestreaming services with goods, they may link this emotional hypocrisy to the poor quality of goods when they perceive surface acting. If the user anticipates a possible negative outcome, they will choose an action strategy to avoid such an outcome (i.e., not to use the livestreaming service again). Therefore, for the users of prevention focus, surface acting makes them generate lower immersion experience and user stickiness. When perceiving deep acting, as the anchor emotional expression is a real state generated from the inside out, the users of prevention focus do not find cues related to low security and therefore release the judgment related to risk. After the security needs are realized, the intrinsic motivation of users to stay continuously is strengthened, resulting in higher immersion experience and user stickiness.

Zhang et al. (2018) showed that individuals with promotion focus possess more positive attitudes toward hedonic gratification and are more sensitive to the hedonic attributes of a product or service. Users with promotion focus were more concerned with hedonic and attractiveness-related characteristics (e.g., whether the anchor was passionate and funny). They differentiate to a lesser extent between the perceptions of the two emotional labor strategies and are more inclusive of emotions (Yeo and Park, 2006). They are more inclusive of emotions and do not distinguish much between the two emotional labor strategies (Winterheld and Simpson, 2011). Users with promotion focus will dig deeper into the hedonic value utility of emotional labor, rather than thinking in terms of self-protection (Song and Qu, 2018). Therefore, the difference between the perceived effects of the two emotional labor strategies on the immersion experience and user stickiness is not significant. Based on the above-mentioned analysis, the hypothesis is proposed as follows:

H4: Regulatory focus moderates the effect of emotional labor (surface acting vs. deep acting) on immersion experience and user stickiness. Specifically, the effect of emotional labor (surface acting vs. deep acting) produced a more significant effect on the users with prevention focus compared with promotion focus.

**Figure 1:**
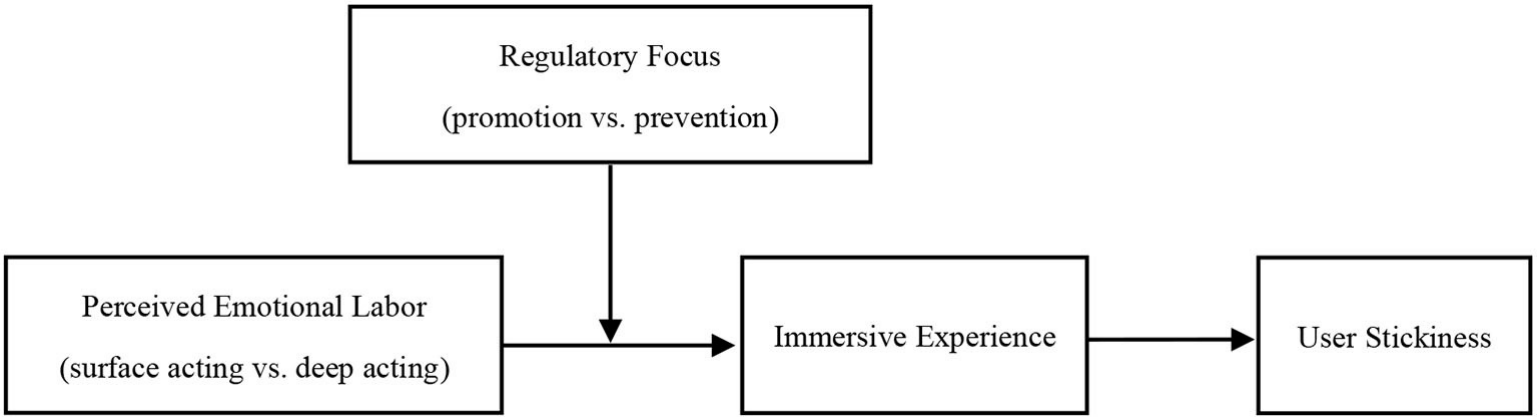
Conceptual framework.

In summary, the theoretical model is shown in Figure 1.

## Hypothesis Testing

Statistics from China Internet Network Information Center (CNNIC) show that livestreaming users in China had reached 560 million by March 2020. The data for this study were collected from livestreaming users in China, which are very representative.

Three studies were designed to test these hypotheses. The questionnaire method was used to measure variables to examine the main effect of emotional labor (hypothesis H1) and the mediating role of the immersion experience (hypothesis H3) in Study 1. The experimental method was used to simulate livestreaming scenarios in Study 2 and Study 3. We stimulated and manipulated the subjects with experimental materials such as text and video, and we observed the differential effects of different emotional labor (i.e., surface acting vs. deep acting) with different regulatory focus (i.e., preventive focus vs. promotion focus) on user stickiness. Study 2 was designed to test the hypothesis H2, and Study 3 was designed to test the hypothesis H4.

### Study 1

#### Study Design

This study collected the data with the help of an online questionnaire platform and selected users from a livestreaming virtual community as the survey object. We contacted two influential opinion leaders in this virtual community and completed the questionnaire distribution and collection process with their assistance.

The first part of the questionnaire is a short description, including the purpose of the study and the commitment to confidentiality. Then, respondents were asked to recall an experience of watching a livestreaming and some details (e.g., some characteristics of the network anchor). Later, they were asked to fill in a scale based on their recollections, including the evaluation of the perception of the emotional labor of network anchor, immersion experience, and user stickiness. In the final part of the questionnaire, respondents were asked to fill in demographic information.

A total of 382 questionnaires were collected, and 305 valid questionnaires remained after excluding invalid questionnaires, with an effective rate of 79.8%. We found that 172 (56.4%) were females and 269 (88.1%) were under the age of 30 years. Referring to previous studies, it basically matches the characteristics of user groups in the livestreaming context.

The measurement of the perceived emotional labor was based on the study of Spencer and Rupp (2009), including four questions such as *I feel that the anchor performs enthusiastically* and *The anchor maintains a pleasant and friendly attitude toward me, The anchor is interested and attentive to me, The anchor remain in a positive mood* (Cronbach's α = 0.81). The measurement of the immersion experience was based on the study of Fang et al. (2019), including four questions such as *I often forget about other things while watching livestreaming I often find that time passes quickly while watching livestreaming. I could not notice what was going on around me while watching livestreaming. My attention was completely focused on it while watching livestreaming*. (Cronbach's α = 0.82). The measurement of the user stickiness was based on the study of Tsao (2014), including three questions such as *I would like to watch this livestreaming again in the future I would be happy to watch this livestreaming. I would like to stay here for a long time* (Cronbach's α = 0.79).

#### Common Method Bias

The issue of common method bias may have disruptive effects in the self-reported questionnaires. The following study is designed to minimize these effects. First, to ensure that the subjects understood the questions accurately, we conducted a small-scale pre-study and adjusted some of the questions. Second, the questionnaire was conducted anonymously, with text indicating to the subjects that the data were only for academic research. Finally, we used the Harman's single factor method for the common method bias test. The results showed that the maximum principal component factor explains a total of 30.93% of the variance (i.e., less than the 40% threshold), so it can be assumed that there is no serious effect of common method bias.

#### Descriptive Statistical Analysis and Validity Test

The variables and related indicators are shown in Table 1. Perceived emotional labor is positively correlated with immersion experience (*r* = 0.39, *p* < 0.01) and user stickiness (*r* = 0.40, *p* < 0.01). The immersion experience is positively correlated with user stickiness (*r* = 0.39, *p* < 0.01). The CR values of all variables were above 0.84, indicating good reliability of the combination. The AVE values of all variables were above 0.58, indicating good convergent validity.

**Table 1:** Descriptive statistical analysis of variables.

**Variables**	***M***	**SD**	**AVE**	**CR**	**1**	**2**	**3**
1. Perceived emotional labor	3.11	1.13	0.58	0.84	(0.76)		
2. Immersion experience	2.86	1.11	0.60	0.86	0.39^**^	(0.77)	
3. User stickiness	3.32	1.12	0.69	0.87	0.40^**^	0.39^**^	(0.83)

** *p < 0.01; The value on the diagonal is the square root of the AVE value*.

The AMOS was used for model fitting to test the structural validity, and the fitting results are shown in Table 2. The three-factor measurement model fit was the best, and all indicators met the criteria (χ^2^*/df* = 4.97, *CFI* = 0.92 > 0.90, *IFI* = 0.92 > 0.90, *NFI* = 0.91 > 0.90, *RMSEA* = 0.05 < 0.06). The study model can be considered to have good structural validity.

**Table 2:** Test of structural validity.

**Model**	**Combinations of factors**	**χ^2^/*df***	**CFI**	**IFI**	**NFI**	**RMSEA**
Three-factor model	PEL; IE; US	4.97	0.92	0.92	0.91	0.05
Two-factor model	PEL+IE; US	13.94	0.74	0.75	0.73	0.16
Two-factor model	PEL; IE+US	17.31	0.68	0.67	0.67	0.23
One-factor model	PEL+IE+US	25.24	0.51	0.52	0.51	0.28

#### Analysis of Results

The SPSS macro plugin PROCESS v3.3 was used to examine the hypotheses H1 and H3 (Hayes, 2013). Confidence interval was set to 95, and sample size was set to 5,000. The results run under Model4 are shown in Table 3. Perceived emotional labor positively affected immersion experience (β = 0.39, *SE* = 0.05, *p* < 0.01) and user stickiness (β = 0.30, *SE* = 0.06, *p* < 0.01). Immersion experience positively affected user stickiness (β = 0.29, *SE* = 0.06, *p* < 0.01). The effect value of perceived emotional labor on user stickiness is 0.30 (*LLCI* = 0.1878 and *ULCI* = 0.4158), and the effect value of perceived emotional labor mediated through immersion experience on user stickiness is 0.11 (*LLCI* = 0.0612 and *ULCI* = 0.1759). Therefore, the hypotheses H1 and H3 were verified.

**Table 3:** Bootstrap effect analysis.

**Effect path**	**Effect size**	**SE**	**LLCI**	**ULCI**
Direct effect Perceived emotional labor → User stickiness	0.30	0.06	0.1878	0.4158
Mediating effect Perceived emotional labor → Immersion experience → User stickiness	0.11	0.03	0.0612	0.1759

Study 1 verified the positive effect of perceived emotional labor on user stickiness and the mediating role of immersion experience by using a questionnaire method. The following Study 2 uses an experimental method to further test the impact of two strategies of emotional labor on user stickiness by simulating a real livestreaming situation.

### Study 2

#### Study Design

Study 2 aimed to examine the effect of two strategies of emotional labor on user stickiness. A one-factor two-level (i.e., surface acting vs. deep acting) between-subjects design was used. The corresponding author has long been engaged in livestreaming service and other related work. A total of 300 volunteers were recruited from several virtual communities and randomly divided into two groups. The total number of questionnaires collected was 203, with a recovery rate of 67.7%. Subjects under the age of 24 years accounted for 86%, and females accounted for 54.09%, basically in line with the distribution characteristics of users in the livestreaming context. After eliminating invalid questionnaires, 183 valid questionnaires were obtained (i.e., 101 in the surface acting group and 82 in the deep acting group), with an effective rate of 90.1%.

First, all subjects were asked to read a textual material, which described the upcoming video as a certain live studio screen to stimulate the sense of presence of subjects in the livestreaming situation. The video showed two different states of emotional labor (i.e., surface acting and deep acting). Referring to the study of Lechner and Paul (2019), we produced the manipulated material with two sets of videos that show a professional actress who was trained in emotional labor techniques. She showed two different states of smiling by modulating her facial muscles. The actress spoke, “Hello everyone, welcome to this live broadcast, the anchor is live every night at 7 p.m., see you soon.” The video was identical in both groups except for differences in the affective state of the actress. The video used in the experiment was recorded using a professional camera (i.e., 1080P, 60FPS) to ensure that it brings the users a sense of presence. Subjects then completed filling in the responses to the dependent variable, the manipulation test, and the demographic variables. Immersion experience was measured with reference to the study of Fang et al. (2019) (Cronbach's α = 0.82), and user stickiness was measured with reference to the study of Tsao (2014) (Cronbach's α = 0.79). The scale used in this study was the 7-point Likert scale.

#### The Test of Validity

The results of the Harman's single factor test using SPSS 21.0 software showed that the first principal component factor explained 29.62% of the variance and can be considered as not having a serious common method bias. In addition, the results of the validation factor analysis showed that the items of same concept were rotated and aggregated together, and all the factor loadings were >0.5 with *KMO* = 0.786 (*p* < 0.01), explaining 69.681% of the total variance. The results of the AMOS model fit showed satisfactory goodness-of-fit indicators (χ^2^*/df* = 2.92, *CFI* = 0.93, *IFI* = 0.92, *NFI* = 0.92, *RMSEA* = 0.05), indicating good discriminant validity of the study model.

#### The Test of Manipulation

Referring to the study of Diefendorff et al. (2005), question items were designed to test the perceptions of emotional labor of the subjects (Cronbach's α = 0.84). After reading the text and video material, subjects were required to evaluate surface acting (e.g., *I think the anchor is hiding his true mood and feelings*) or deep acting (e.g., *I think the anchor is genuinely expressing her feelings rather than disguising them*). The results showed that the experiment successfully manipulated the perception of emotional labor. Specifically, subjects in the surface acting group had significantly higher perceived surface acting scores (*M* = 4.77) than those in the deep acting group (*M* = 3.96, *F* = 14.326, *p* < 0.05). As for subjects in the deep acting group, their perceived deep acting scores (*M* = 4.57) were significantly higher than those in the surface acting group (*M* = 3.82, *F* = 8.769, *p* < 0.05).

#### Analysis of Results

**Figure 2:**
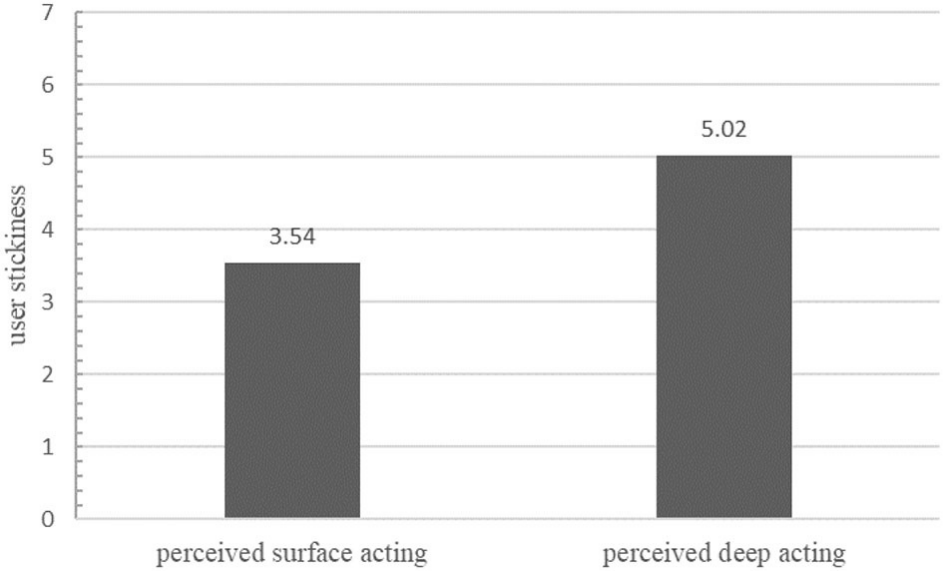
Comparison of differences in user stickiness under differently perceived emotional labor.

We used the ANOVA method to test the main effects with user stickiness as the dependent variable and perceived emotional labor as the independent variable. The results showed a significant difference in the effect of the two emotional labor strategies on user stickiness (*F* = 53.203, *p* < 0.01), with a significantly higher mean value of user stickiness in the deep performance group (*M* = 5.02 > 3.54). A comparison of the differences in user stickiness is shown in Figure 2. Thus, deep acting in emotional labor has a stronger positive impact on user stickiness than surface acting, and the hypothesis H2 was verified.

The plugin PROCESS was used to perform the robustness tests on the hypothesis H3. The bootstrap test was performed under the condition of 5,000 sample sizes and 95% CIs. The results showed that the mediating effect of immersion experience was significant (*LLCI* = −0.3072 and *ULCI* = −0.0800). The hypothesis H3 was again verified. Then, a second experiment (i.e., Study 3) was designed to examine the hypothesis H4.

### Study 3

#### Study Design

Study 3 aimed to examine the moderating effect of regulatory focus, using a 2 (emotional labor: surface acting vs. deep acting) × 2 (regulatory focus: prevention focus vs. promotion focus) between-group design. Volunteers from several virtual communities were recruited and randomly divided into four groups. Finally, 248 questionnaires were collected, and 235 valid questionnaires were obtained after eliminating invalid questionnaires, with an effective rate of 94.7%.

The task-initiated method used in this study activated the regulatory focus of subjects. Subjects were required to complete a word-selection task (i.e., selecting words that fit the requirements from a word matrix consisting of 5 × 5). In the promotion focus, subjects were asked to select words related to *benefit* (e.g., pleasure and satisfaction) from the given word matrix and then to write down two expectations for the future. In the prevention focus, subjects were asked to select words related to *risk* (e.g., cheat and fake) from the same word matrix and then to write down two obligations. Later, subjects were asked to complete the same content as in Study 2.

#### Tests of Validity and Manipulation

The results of the factor analysis showed that the first principal component factor explained 22.059% of the variance and can be considered as not having a serious common method bias. The validity of the study also qualified (*KMO* = 0.757, *p* < 0.01).

A bipolar 7-point scale was used for the manipulation test of regulatory focus, where subjects chose the viewpoint that was relatively more consistent at the left and right ends of the scale (e.g., 1 = *I would prefer to do what everyone agrees is right* and 7 = *I would prefer to do what I want to do*). Low scores represent individuals belonging to prevention focus. The results indicate that the experiment successfully manipulated the perception of regulatory focus. Subjects in the promotion focus group scored significantly higher (*M* = 5.29) than those in the prevention focus group (*M* = 3.27, *F* = 144.395, *p* < 0.01).

The manipulation test for perceived emotional labor was the same as in Study 2. Subjects in the surface acting group had a significantly higher perceived surface acting score (*M* = 4.68) than those in deep acting group (*M* = 3.51, *F* = 47.946, *p* < 0.01). Subjects in the deep acting group had a significantly higher perceived deep acting score (*M* = 4.50) than those in surface acting group (*M* = 3.97, *F* = 68.832, *p* < 0.01). Thus, the perception of emotional labor was successfully manipulated.

#### Analysis of Results

**Figure 3:**
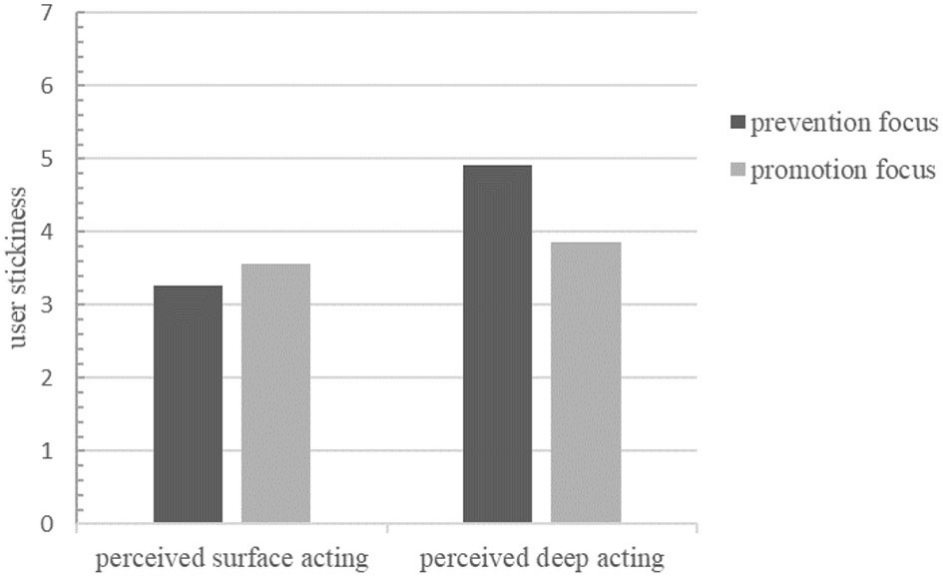
Comparison of user stickiness with different regulatory focus.

The results of the ANOVA showed that the main effect of emotional labor on user stickiness was significant (*F* = 68.832, *p* < 0.01), the main effect of regulatory focus on user stickiness was significant (*F* = 21.375, *p* < 0.001), and the interaction between emotional labor and regulatory focus was significant for user stickiness (*F* = 2.866, *p* < 0.05). The difference in user stickiness between groups for users with prevention focus was significant (*F* = 15.744, *p* < 0.01) and for those with promotion focus was not significant (*F* = 1.171, *p* > 0.05). A comparison of the differences in user stickiness is shown in Figure 3.

**Figure 4:**
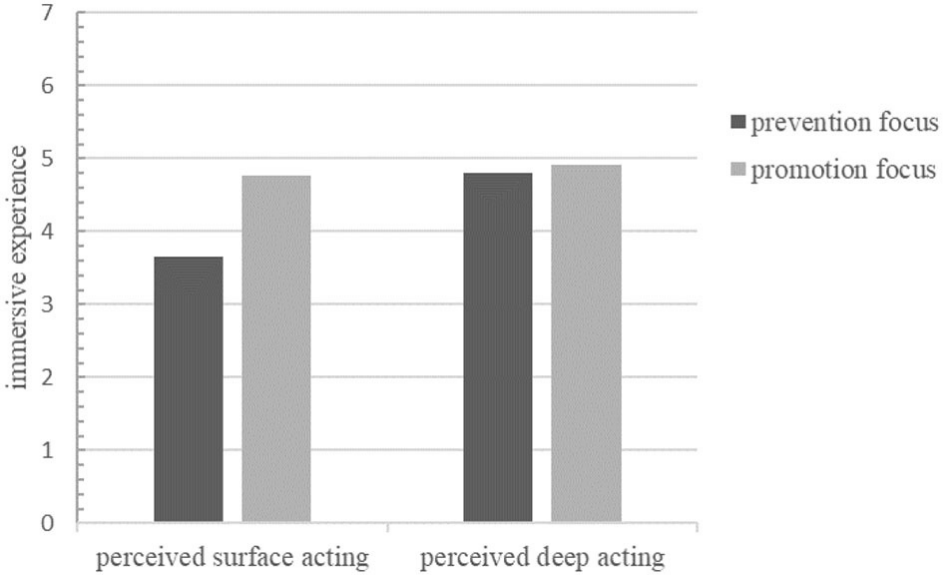
Comparison of immersive experience with different regulatory focus.

We also analyzed the differential impact of immersion experience under different regulatory focus. Perceived emotional labor (*F* = 21.432, *p* < 0.01) and regulatory focus (*F* = 13.834, *p* < 0.01) significantly affected the immersion experience, as did the interaction (*F* = 4.191, *p* < 0.05). The difference in immersion experience between groups for users with prevention focus was significant (*F* = 19.128, *p* < 0.01) and for those with promotion focus was not significant (*F* = 0.983, *p* > 0.05). In summary, the hypothesis H4 was verified. A comparison of the differences in immersion experience was shown in Figure 4.

## Discussion

All the hypotheses proposed in this study have been verified, and the results include the following points. First, emotional labor positively influenced user stickiness. Second, compared with surface acting, deep acting has a stronger effect on user stickiness. Third, immersion experience plays a mediating role between emotional labor and user stickiness. Fourth, regulatory focus moderates the positive effect of emotional labor on user stickiness. Specifically, users with prevention focus generate lower user stickiness when perceiving surface acting and higher user stickiness when perceiving deep acting. Both emotional labor strategies result in high user stickiness for users with promotion focus.

The theoretical contributions of this study are as follows: First, there are few studies on user stickiness in the context of livestreaming service, and this study bridges this theoretical gap. Second, previous studies on user stickiness have paid less attention to the influence of affective factors. This study enriches the related research by exploring the role of emotional labor in the formation process of user stickiness. Third, unlike previous studies that focus on service providers, this study chooses the perspective of service recipient (i.e., user), which expands the perspective of emotional labor research. This also echoes the call of some scholars for focusing on the perceptual perspective of service recipient (Groth et al., 2009; Liu et al., 2019; Gong et al., 2020). Fourth, the importance of the immersion experience of user in the context of livestreaming service is highlighted. Fifth, the differential role of different regulatory focus users is examined, broadening the theoretical boundaries.

The study findings provide rich practical insights for livestreaming service practitioners. First, the role of the emotional labor of network anchors in improving user stickiness needs to be emphasized. Anchors can take steps to stimulate the perception of emotional labor of users (e.g., by participating in bullet–screen interactions and interacting with users in real time). Anchors should pay attention to their own emotional expressions and strategic choices (i.e., deep acting). Second, appropriate ways should be chosen to improve the immersion experience of the user. Anchors are expected to use deep acting for emotional labor, which greatly benefits the generation immersion experience. Activities can be held to stimulate the willingness of users to make bullet–screen comments, which is beneficial to the immersion experience of users. Finally, the different regulatory focus of the user group should be fully explored and the targeted services should be taken. For example, more considerations should be given to service strategies that increase hedonic value for users with promotion focus, and the professionalism and reliability of the livestreaming style should be given more attention for users with prevention focus. Different styles of anchors can be recommended to users with different regulation focus, improving the accuracy of livestreaming services and enhancing user stickiness.

As with all research, this study has several limitations. First, the sample of the senior part in all three studies is small, although it is consistent with the age distribution characteristics of the user group in the context of livestreaming service. Second, we focused on user stickiness in the context of livestreaming services, the generalizability to other online service industries needs to be further examined. Finally, future research can continue to focus on the role of emotional factors in improving user stickiness and find more ways to enhance user stickiness.

## Data Availability Statement

The datasets generated for this study are available on request to the corresponding author.

## Author Contributions

YS developed the study design and drafted the manuscript. CM coordinated the data collection and computed the analyses. YZ contributed to data collection, data interpretation, and commented on the first draft of the manuscript. All the authors approved the final version of the manuscript.

## Conflict of Interest

The authors declare that the research was conducted in the absence of any commercial or financial relationships that could be construed as a potential conflict of interest.
